# Interfacial Regulation by a NiO*_x_* Overlayer Enables Enhanced Near‐Infrared Photoelectrochemical Water Splitting

**DOI:** 10.1002/smsc.70346

**Published:** 2026-07-23

**Authors:** Xiao‐Feng Shen, Kyle J. Stephens, Dengyao Yang, Nick A. Shepelin, Kuan‐Ting Wu, Kazuto Hatakeyama, Daniele Pergolesi, Shintaro Ida, Motonori Watanabe, Thomas Lippert

**Affiliations:** ^1^ International Institute for Carbon–Neutral Energy Research Kyushu University Fukuoka Japan; ^2^ Center for Neutron and Muon Sciences Paul Scherrer Institute Villigen Switzerland; ^3^ Department of Chemistry and Applied Biosciences ETH Zürich Zürich Switzerland; ^4^ Institute of Industrial Nanomaterials (IINa) Kumamoto University Kumamoto Japan; ^5^ PSI Center for Energy and Environmental Sciences Paul Scherrer Institute Villigen Switzerland; ^6^ Hydrogen Institute for Sustainability Kyushu University Fukuoka Japan

**Keywords:** low‐temperature pulsed laser deposition, near‐infrared dye sensitizer, photoelectrochemical reaction

## Abstract

Photoelectrochemical (PEC) water splitting provides a promising pathway for sustainable hydrogen production. However, the inefficient use of near‐infrared (NIR) light, which accounts for nearly half of the solar spectrum, remains a major limitation to overall energy conversion efficiency. Here, we present a NIR‐responsive dye‐sensitized photoanode enabled by a boron–dipyrromethene–carbazole‐based organic sensitizer (**1**), combined with a thin NiO_*x*_ overlayer that acts as both an oxygen evolution reaction (OER) cocatalyst and an interface regulator. The NiO_*x*_ layer, deposited via low‐temperature pulsed laser deposition, exhibits low crystallinity and mixed Ni^2+^/Ni^3+^ valence states, as confirmed by transmission electron microscopy and X‐ray photoelectron spectroscopy. Adding the NiO_*x*_ overlayer results in a 1.9‐fold increase in photocurrent density and significantly better photostability. Electrochemical testing indicates that NiO_*x*_ alters interfacial charge–transfer kinetics and the local electrochemical environment, thereby improving carrier utilization and reaction efficiency. The multilayer photoanode retains a measurable photocurrent response at 850 nm, demonstrating its capability to extend PEC activity into the NIR region. This work elucidates the synergistic functions of NiO_*x*_ in interfacial charge regulation and catalytic kinetics, offering a viable strategy for NIR‐driven solar fuel conversion. Additionally, it establishes the longest‐wavelength photoelectrocatalytic performance reported to date for non‐noble‐metal dye‐sensitized systems.

## Graphical abstract

A NiO_*x*_‐modified dye‐sensitized photoanode enabled near‐infrared‐driven photoelectrochemical water splitting. The NiO_*x*_ overlayer promoted hole extraction, accelerated oxygen evolution, and stabilized the interface. This interfacial engineering strategy enhanced activity and stability, providing an effective pathway for extending solar fuel generation into the near‐infrared region.

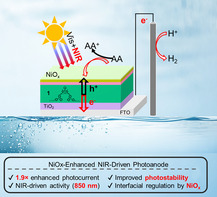

## 1 Introduction

Over the past few decades, energy demand has increased, while fossil resources have been depleted [1, 2, 3, 4, 5, 6, 7]. This has led to an urgent need to reduce annual carbon dioxide emissions, driving a shift toward renewable and environmentally benign energy sources. Solar energy is a widely available, clean, and highly cost‐effective resource that can aid the transition to sustainable energy [8, 9, 10]. Utilizing non‐fossil fuels, such as solar energy, provides an economically viable solution to climate change [11, 12]. In recent years, photocatalysis technologies have demonstrated significant potential for applications in renewable energy conversion and environmental governance [13, 14, 15, 16, 17, 18, 19, 20, 21]. However, traditional photocatalytic materials primarily respond to ultraviolet (UV) and visible light (VIS), resulting in low utilization of near‐infrared (NIR) light. Normally, traditional photocatalytic materials at standard temperature and pressure can only utilize sunlight up to approximately 800 nm, which constitutes approximately 79.0% of the theoretical energy from solar light‐driven hydrolysis to produce hydrogen [16, 22]. This limitation hampers the improvement of photocatalytic efficiency under real sunlight photocatalytic reaction. Although some studies in photocatalytic water splitting reaction have concentrated on developing photosensitized materials that respond to NIR light. Gao et al. [23] reported a CdS/NaYF_4_:Yb^3+^, Er^3+^ up‐conversion photocatalyst that converts 980 nm near‐infrared light into visible light to drive overall water splitting, achieving a hydrogen evolution rate of 3.4 μmol g^−1^ h^−1^ with an apparent quantum efficiency of only 0.008% under NIR irradiation. However, both approaches rely on rare‐earth elements or plasmonic photothermal effects, which limit their scalability and practical applicability. In contrast, organic dye‐sensitized materials have been extensively studied for their ability to absorb various wavelengths of the solar spectrum by modifying their molecular structure [24, 25, 26, 27, 28, 29]. Such as **7c**@Pt–TiO_2_ can use up to 700 nm [29], **2**@Pt–HPT500 can use up to 750 nm [30], **SA1**@Pt–TiO_2_ can use up to 800 nm [24]. Nevertheless, these NIR‐photosensitive materials still face challenges in photocatalytic applications due to their dependence on precious metals and stability of the organic compounds [24, 31, 32]. Therefore, developing novel, high‐efficiency, and environmentally friendly NIR photosensitizing systems remains an urgent challenge.

Additionally, in the photocatalytic water splitting system, the hydrogen production activity is enhanced using co‐catalysts. Among these, the NiO_*x*_ exhibits excellent co‐catalytic properties and chemical stability, thereby enhancing the efficiency of photocatalytic reactions by promoting the segregation and migration of photogenerated holes. However, traditional methods for preparing NiO_*x*_ thin films, such as magnetron sputtering and chemical vapor deposition, are costly and require complex equipment. These methods may lead to problems such as non‐uniform film structure and decomposition of the organic layer, thereby limiting enhancements in photocatalytic performance. In contrast, pulsed laser deposition (PLD) is ideal for producing high‐quality NiO_*x*_ thin films because of its precise control over film composition, structure, and defects [33]. Specifically, the PLD technique enables the growth of semicrystalline phase NiO_*x*_ films at lower temperatures, which prevents thermal damage to the organic films in devices.

**SCHEME 1:**
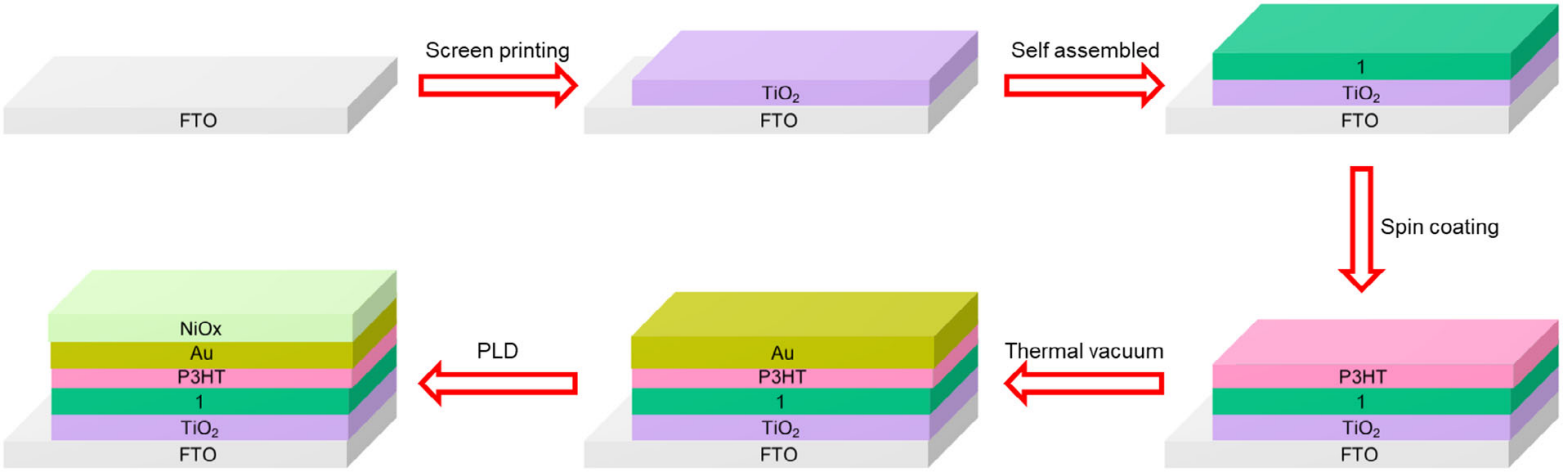
Composition of the working electrode and the deposition methods used for each layer.

To overcome the above challenges, we have designed and synthesized a hybrid photoelectrode comprising a multi‐layer structure (Scheme 1). A metal‐free dye‐sensitizer **1** (shown in Figure 2a), with photon absorption up to 850 nm, is used, coated by PLD with a NiO_*x*_ film with low‐crystallinity phase as a co‐catalyst, to enhance its catalytic activity. The results demonstrate that this organic–inorganic hybrid combination can significantly improve stability, presenting a novel approach to optimizing the performance of the NIR photocatalytic system.

## 2 Results and Discussion

### 2.1 Effect of NiO_x_ Modification on Photoelectrochemical and IPCE Performance

**FIGURE 1:**
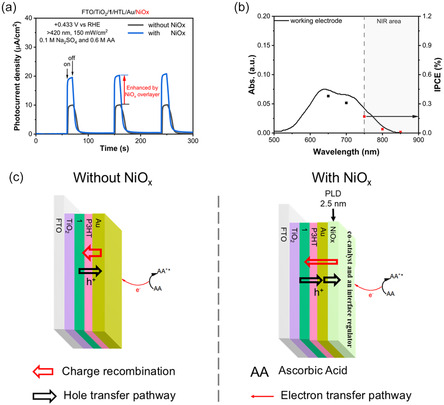
(a) Photocurrent measurement results of NiO_*x*_‐modified and unmodified electrodes (>420 nm, 150 mW/cm^2^, pH4.0, 0.1 M Na_2_SO_4_, and 0.6 M ascorbic acid); (b) the IPCE results of FTO/TiO_2_/1/HTL/Au/NiO_*x*_ (pH4.0, 0.1 M Na_2_SO_4_, and 0.6 M ascorbic acid); (c) proposed mechanisms for electrode transfer in unmodified and modified NiO_*x*_ electrodes.

Upon light irradiation, a rapid and stable photocurrent is generated, while the photocurrent promptly drops to nearly zero once the light is switched off. The unmodified electrode exhibits a photocurrent density of 10.1 μA/cm^2^, whereas the NiO_*x*_‐modified electrode delivers a significantly enhanced value of 19.6 μA/cm^2^, corresponding to a 1.9‐fold increase (Figure 1a). Notably, the photocurrent response demonstrates excellent reproducibility over repeated on–off cycles. To further verify the photoresponsivity of FTO/TiO_2_/**1**/hole transfer layer (HTL)/Au/NiO_*x*_ in the near‐infrared region, an incident photon‐to‐current conversion efficiency (IPCE) test was conducted in the 650–850 nm range. The results, shown in Figure 1b, indicated that the overall trend of the IPCE curves was consistent with the diffuse reflectance spectroscopy (DRS) spectrum of the working electrode, suggesting a good synergistic relationship between its light‐absorbing behavior and photoelectric conversion performance. Furthermore, the NiO_*x*_‐modified electrode maintains IPCE values of approximately 0.17% at 750 nm, 0.04% at 800 nm, and 0.01% at 850 nm, demonstrating its potential to extend PEC activity into the NIR region. As summarized in Figure 1c, the surface is coated with a thin layer of NiO_*x*_, which not only acts as a cocatalyst for the oxygen evolution reaction (OER) but also serves as an interfacial modifier, leading to an increase in charge separation time and a decrease in recombination probability, which ultimately enhances overall activity performance.

### 2.2 NIR‐Responsive Photophysical Properties of the Dye Sensitizer

**FIGURE 2:**
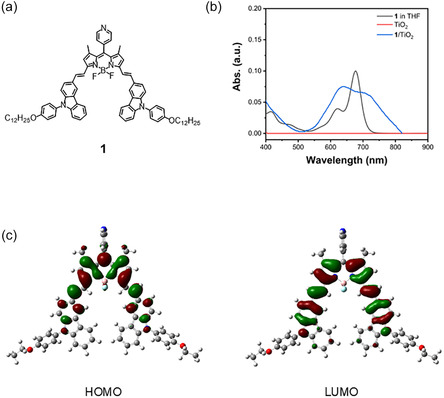
(a) Structure and fully optimized HOMO–LUMO of **1**; (b) absorption spectrum of **1** in THF solution, diffuse reflectance absorption spectra of TiO_2_ and **1**/TiO_2_; (c) Optimized HOMO–LUMO of **1**.

The molecular structure of boron–dipyrromethene (BODIPY)‐sensitizer (**1**), shown in Figure 2a, serves as the basis for the subsequent discussion. The UV–vis absorption spectrum of **1** in THF solution (Figure 2b) exhibits an absorption maximum at 676 nm with a high molar extinction coefficient (*ε*) of 360 000 and a broad absorption band between 400 and 520 nm (black line in Figure 2b). With an absorption band edge at 726 nm, the bandgap of **1** was calculated to be 1.67 eV using the de Broglie formula. Compared with the DRS spectrum of TiO_2_ (red line in Figure 2b), a new absorption band appears in the range of 500–850 nm after **1** loading. As a result, the **1**/TiO_2_ sample exhibits visible‐to‐near‐infrared light absorption, with a maximum at 709 nm (blue line in Figure 2b). Compared with the spectrum of **1** in THF solution, the absorption maximum was redshifted, indicative of *J‐*type aggregation of the dye molecules on the TiO_2_ surface [34]. In the ATR–IR spectra, a characteristic Ti—OH vibration was observed at 1647 cm^−1^ for the TiO_2_ film. After dye‐loading, this peak disappeared and shifted to 1635 cm^−1^, strongly suggesting Lewis's acid‐type Ti—N coordination occurred between dye **1** and TiO_2_ (Figure S1). Based on DRS and IR spectroscopy, dye **1** is loaded at the Ti site of TiO_2_, and the molecules interact with each other. Figure 2c shows the highest occupied molecular orbital (HOMO) and the lowest occupied molecular orbital (LUMO) orbitals of **1**. The HOMO and LUMO orbitals are delocalized from the BODIPY core to the extended carbazole aromatic moieties, showing significant spatial overlap. This orbital overlap gives rise to a broad absorption band in the range of 600–750 nm, which can be attributed to an intramolecular π–π transition* [35].

### 2.3 Physicochemical Characterization of the NiO_x_ Overlayer

**FIGURE 3:**
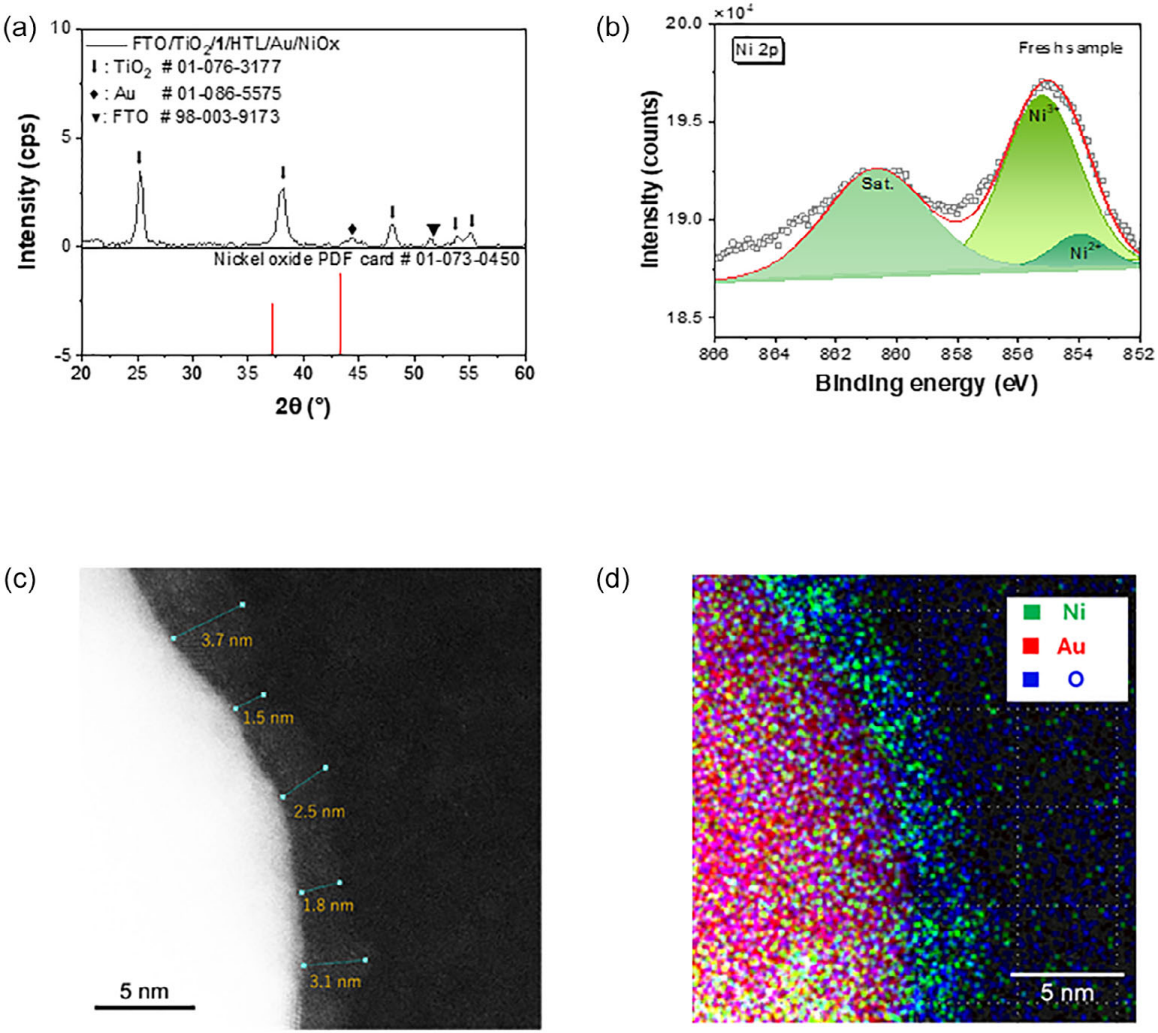
Basic physical properties of the electrode: (a) The XRD result; (b) the XPS result; (c) the TEM image; (d) the TEM–EDX image.

To improve catalytic activity, NiO_*x*_ was selected as a co‐catalyst due to its favorable redox properties and earth‐abundant nature. A conformal NiO_*x*_ layer was then deposited through PLD, allowing precise control of thickness and ensuring close contact at the interface. As shown in Figure 3a, the peaks belonging to anatase TiO_2_ are observed at 2θ values corresponding to 25°, corresponding to the (101) reflection, 38°, corresponding to the (004) reflection, 48°, corresponding to the (200) reflection, 54°, corresponding to the (105) reflection, and 55°, corresponding to the (211) reflection. Additional peaks are observed, which are assigned as Au at 38°, corresponding to the (111) reflection and at 44°, corresponding to the (220) reflection, as well as FTO at 52°, corresponding to the (111) reflection. No peak representative of NiO_*x*_ was observed. Even for the 34.6 nm NiO_*x*_ deposited on a MgO substrate at 300°C, only the peak at 43.1°, corresponding to the (002) plane of MgO, was observed (Figure S2), suggesting a low crystallinity or as crystals with a Nanometer**–**sized dimension of NiO_*x*_ by this study of PLD deposition condition. To ascertain the chemical composition and oxidation state of the NiO_*x*_ layer, XPS analysis was conducted. The Ni2p 3/2 XPS spectrum of the FTO/TiO_2_/**1**/HTL/Au/NiO_*x*_ electrode is shown in Figure 3b. NiO_*x*_ is bivalent, consisting of a mixture of Ni^2+^ and Ni^3+^ ions. The spectral peaks observed at 854.0 and 855 eV are ascribed to Ni^2+^ and Ni^3+^ ions, with respective area percentages of 11.8% and 88.2% [36, 37]. Furthermore, the peak observed at 860.9 eV corresponds to the Ni 2p 3/2 shake‐up satellite. The SEM images confirmed that the electrode surface is coated with TiO_2_ films, HTL, and Au with layered structures; however, due to the extremely thin layers of NiO_*x*_, distinct interfaces cannot be observed (Figure S3). The TEM image shown in Figure 3c confirms the presence of a NiO_*x*_ layer on the surface. This layer was found to continuously cover the Au layer without detectable gaps or discontinuities, indicating uniform, conformal coverage. TEM analysis revealed a NiO_*x*_ layer thickness of 1.5–3.7 nm, with an average thickness of approximately 2.5 nm. This also suggests that the deposition of an ultrathin coating was successful with an approximate PLD deposition rate of 4.8 Å/s. Figure 3d shows the TEM–EDX mapping of the electrode, which further reveals a uniform distribution of Ni over the Au surface with a thickness of approximately 2.5 nm. In addition, TEM observations revealed the presence of localized crystalline domains, and lattice fringe analysis identified planes corresponding to the (111) plane of NiO (Figure S4). Although no distinct crystalline diffraction peaks were detected in the XRD patterns (Figure 3a), the TEM results suggest that the presence of lattice fringes with a spacing corresponding to the (111) plane of NiO suggests the existence of locally crystalline domains, confirming the partially crystalline nature of the NiO_*x*_ layer.

### 2.4 Synergistic Effects of Electrode Design and Electrolyte Composition

**FIGURE 4:**
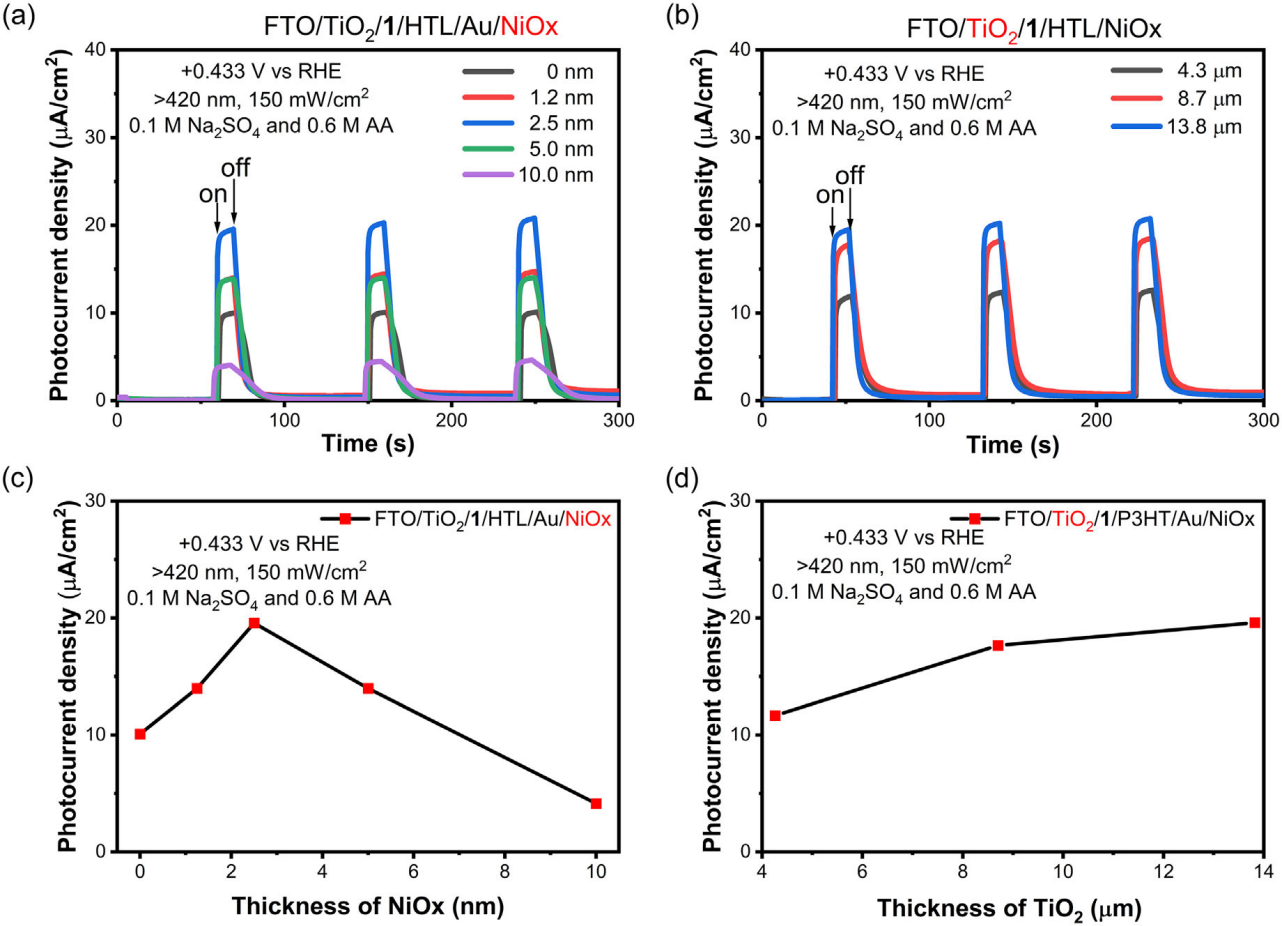
(a) Photocurrent density of different PLD deposition time samples; (b) photocurrent density of different TiO_2_ thickness samples; (c) the relationship between the PLD deposition time and photocurrent performances; (d) the relationship between TiO_2_ thickness and photocurrent performances.

First, the NiO_*x*_ deposition time was varied to achieve different thicknesses of NiO_*x*_ films (0 nm, 1.2, 2.5, 5.0, and 10.0 nm, respectively). Photocurrent response measurements were performed in 0.1 M Na_2_SO_4_ and 0.6 M ascorbic acid solutions under visible light irradiation (>420 nm). As shown in Figure 4a, in a chronoamperometry photocurrent–time (I–t) response under intermittent illumination, the photocurrent is enhanced under illumination with the additional NiO_*x*_ layer, suggesting that NiO_*x*_ enhances the photoanodic reaction. Additionally, when the light is turned off, the photocurrent immediately drops to zero, indicating no dark current leakage/ dark OER occurs in NiO_*x*_‐deposited electrodes. Moreover, good repeatability of the light response was observed. The maximum current densities for each condition were selected and then plotted in Figure 4c. Compared to the unmodified electrode (10.1 μA/cm^2^), the photocurrent density exhibits a volcano‐like behavior and reaches a maximum at 2.5 nm (19.6 μA/cm^2^). Beyond 2.5 nm, the photocurrent density decreased with increasing thickness, reaching 14.0 μA/cm^2^ for 5 nm. When the thickness reached 10.0 nm (4.1 μA/cm^2^), the photocurrent density fell below that of the unmodified electrode. Notably, the optimal performance of the 2.5 nm NiO_*x*_ layer is not limited to the organic–inorganic hybrid photoelectrode studied here. In our recent work, a similar NiO_*x*_ thickness (2.5 nm) on oxynitride films also exhibited the highest photoelectrochemical efficiency [38]. The NiO_*x*_ films also showed dependence on other electrode parameters; therefore, the TiO_2_ layer thickness was optimized. A good reproducibility of the photocurrent density is shown in Figure 4b, indicating stable device performance. As shown in Figure 4d, with the thickness of the TiO_2_ layer increased, the photocurrent density showed an upward trend from the initial 11.7 μA/cm^2^ (4.3 μm TiO_2_) to 17.7 μA/cm^2^ (8.7 μm TiO_2_) and peaked at 19.6 μA/cm^2^ (13.8 μm TiO_2_). Kang et al. reported that the dye‐loaded TiO_2_ film exhibits a maximum photocurrent density when its thickness is around 15 µm [39]. This behavior can be rationalized by the interplay between light harvesting and charge transport. Limited dye loading restricts photon absorption in thin films, whereas extended transport pathways in thicker films increase recombination and diffusion losses, thereby lowering the photocurrent. However, attempts to further increase the TiO_2_ layer thickness resulted in an inability to form a continuous TiO_2_ film layer during sintering due to an excessively thick precursor coating (Figure S5).

**FIGURE 5:**
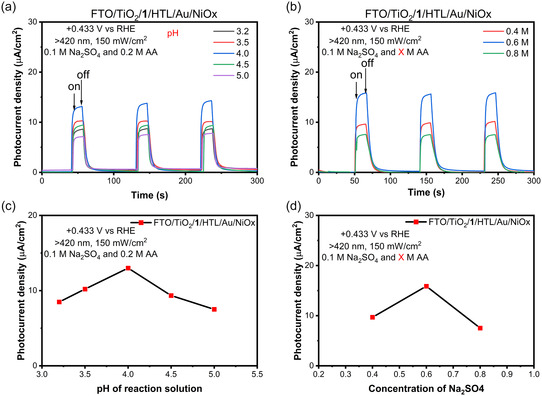
(a) Photocurrent density of different pH; (b) photocurrent density of different ascorbic acid concentrations; (c) the relationship between pH and photocurrent performances; (d) the relationship between ascorbic acid concentrations and photocurrent performances.

To further optimize the photocatalytic reaction conditions, the parameters of the reaction solution were also optimized. Among these, pH was identified as a critical factor that significantly affects PEC performance and is thermodynamically more favorable under acidic conditions. The photocurrent response in the pH range 3.2–5.0 was evaluated [30]. As shown in Figure 5a, the photocurrent density remained relatively low (8.5 μA/cm^2^) in highly acidic conditions (pH 3.2). As the pH increased, the photocurrent density increased, reaching a maximum at pH 4.0 (16.0 μA/cm^2^). However, with further increases in pH to 5.0, the photocurrent density decreased to 7.5 μA/cm^2^. According to Ruiz et al. [40], pH 4 lies in the optimal mechanistic window (pH 2–5) where two protons participate in the stepwise two‐electron oxidation, allowing the rate‐determining electron‐transfer step to proceed more rapidly. In contrast, strong acidity at pH 2 disturbs the acid–base equilibrium, while pH 6 falls into the one‐proton regime where the oxidation mechanism changes and reversibility deteriorates. As shown in Figure 5c, this bell‐shaped trend is consistent with prior studies, which report that performance is strongly pH‐dependent in photocatalytic reactions [41, 42]. Moreover, the ascorbic acid concentration, serving as a sacrificial agent, was also optimized (Figure 5b,d). The measured photocurrent densities were similar at 0.4 M (9.7 μA/cm^2^) and 0.8 M (7.5 μA/cm^2^) concentrations, with the highest photocurrent density recorded at 0.6 M (15.9 μA/cm^2^). This finding indicates that a moderate concentration of the sacrificial agent promotes more effective removal of photogenerated holes while mitigating the potential surface adsorption inhibition that can arise from excessively high concentrations. Finally, the concentration of Na_2_SO_4_ was also optimized. The photocurrent densities of 0.1, 0.2, and 0.3 M were 13.8, 12.7, and 12.6 μA/cm^2^. The results indicated that increasing the concentration of Na_2_SO_4_ had little effect on the photocurrent efficiency (Figure S6).

### 2.5 Charge Transfer and Photocurrent Correlation

To further elucidate the impact of NiO_*x*_ thickness on charge transfer from the dye sensitizer, electrochemical impedance spectroscopy (EIS) measurements were performed under both dark and light‐illuminated conditions for electrodes with different NiO_*x*_ thicknesses (Figure S7).

**FIGURE 6:**
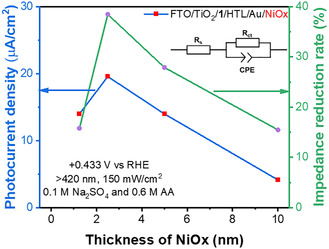
Relationship between photocurrent enhancement and interfacial charge‐transfer improvement for electrodes with different NiO_*x*_ thicknesses.

The results show that the overall impedance gradually increases with increasing NiO_*x*_ thickness, indicating that excessively thick NiO_*x*_ layers introduce additional resistance to charge transport (Table S1). However, when comparing the impedance reduction upon illumination, the 2.5 nm NiO_*x*_ electrode exhibits the largest decrease in charge–transfer resistance among all samples. Importantly, as shown in Figure 6, both the photocurrent density and impedance reduction rate exhibit a volcano‐type dependence on NiO_*x*_ thickness, reaching maximum values at 2.5 nm. A similar optimum‐thickness behavior has been reported for NiO_*x*_‐modified oxynitride photoanodes, where a 2.5 nm NiO_*x*_ overlayer maximized the photocurrent density, indicating that excessive NiO_*x*_ loading can be detrimental despite its catalytic function [38]. Further increasing the NiO_*x*_ thickness results in a gradual decline in both photocurrent and the rate of impedance reduction. Notably, if the primary role of NiO_*x*_ were solely to provide additional catalytic active sites, the photocurrent would be expected to increase, or at least saturate, with increasing NiOx loading within the ultrathin regime. However, the observed decrease in photocurrent at larger thickness indicates that excessive NiO_*x*_ introduces additional charge‐transport resistance, which counteracts any potential catalytic benefit. This interpretation is consistent with previous reports showing that NiO_*x*_ can enhance photocurrent primarily by improving interfacial hole injection rather than by modifying bulk charge separation, as evidenced by increased charge‐injection efficiency and reduced charge‐transfer resistance in photoelectrochemical impedance measurements [43]. These results suggest that the dominant contribution of NiO_*x*_ in the present architecture is the regulation of interfacial charge transfer and suppression of charge recombination, while its cocatalytic effect plays a secondary role.

This conclusion is further supported by the thickness‐dependent EIS results and the negligible change in surface morphology observed by AFM measurements (Figure S8), collectively demonstrating that interfacial charge‐transfer optimization is the key factor governing the enhanced performance of the NiO_*x*_‐modified electrodes.

### 2.6 Electrochemical Durability and Photostability of the NiO_x_‐Modified Photoanode

**FIGURE 7:**
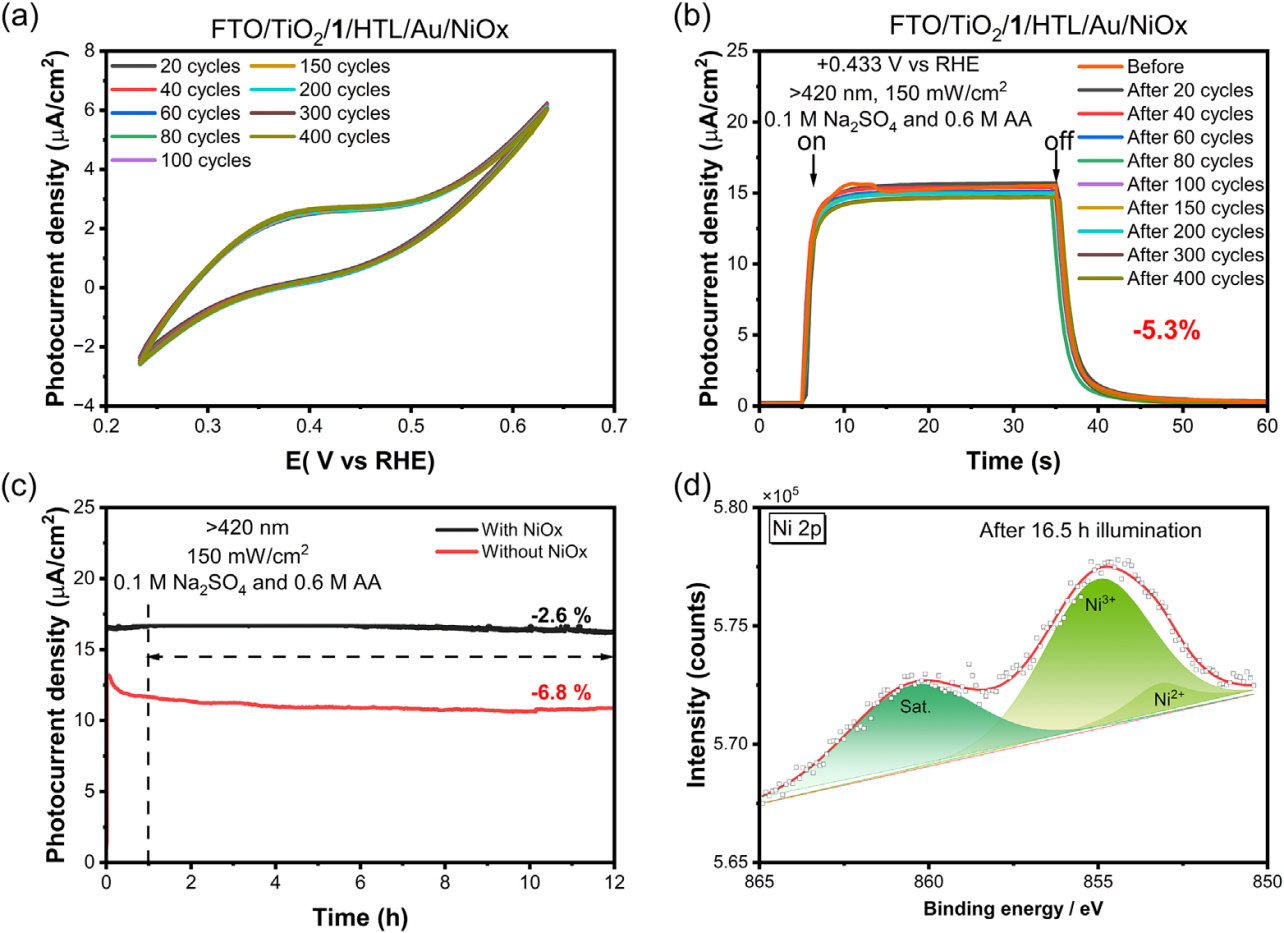
Long‐term electrochemical stability results: (a) CV results; (b) Photocurrent density of each cycle; (c) long‐term light stability results; (d) XPS result of the electrode after 12 h of illumination.

CV measurements were conducted to evaluate the electrochemical stability of the photoelectrode (Figure 7a). Any new peaks appeared within 400 cycles in the range of 0.3–0.7 V versus RHE under dark current conditions, and the intensity of the peaks remained nearly unchanged. Photocurrent tests were performed after 20, 40, 60, 80, 100, 150, 200, 300, and 400 cycles (Figure 7b). Compared to the initial state, only a 5.3% decrease in photocurrent density was observed after 400 cycles of CV. These dark/light current experiments indicated that the electrode was highly stable under our experimental conditions. Meanwhile, the photostability was also measured. The photocurrent density maintained high activity, with only a 3.3% reduction even after 400 cycles of light‐on/off testing (Figure S9).

A control experiment using a FTO/TiO_2_/Au electrode without the dye sensitizer and hole‐transport layer showed no detectable photocurrent under either visible‐light (λ > 420 nm) or NIR (λ > 700 nm) irradiation (Figure S10). This result confirms that the observed photocurrent originates from the dye‐sensitized photoelectrode rather than from the intrinsic photoactivity of the FTO/TiO_2_/Au substrate.

Figure 7c shows the results of continuous illumination. The photocurrent of the unmodified electrode decreased by approximately 6.8% during continuous illumination after removing the transient photocurrent spikes that occurred in the first hour, whereas the NiO_*x*_‐modified electrode exhibited only about a 2.6% decrease. As shown in Figure S11, the NiO_*x*_‐modified electrode retains 95.6% of its initial photocurrent after 16.5 h of continuous operation, demonstrating excellent photoelectrochemical stability. These results demonstrate that the introduction of NiO_*x*_ effectively suppresses performance degradation under illumination, indicating enhanced photostability of the system due to faster charge correction from dye sensitizer and protection of device configuration from water by NiO_*x*_ layer, which is advantageous among current NIR‐responsive photocatalytic materials (Table S2). After 16.5 h of irradiation, the XPS results indicate that Ni^2+^ and Ni^3+^ are also present, with relative areas of 14.8% and 85.2%, respectively (Figure 7d). Compared with the fresh electrode, the area percentages of 11.8% and 88.2% suggest that the valence state of Ni reorganizes during the photocatalytic process, increasing the amount of Ni^2+^. Tian et al. have reported that Ni^3+^ can be reduced to Ni^2+^ through thermal reduction [44]. In addition, Ni^3+^ ions can be reduced to Ni^2+^ ions by the electrons lost by the oxidation of glucose to gluconolactone [45]. Furthermore, ascorbic acid has a strong capability to provide electrons, so it not only acts as an electron donor in the photocatalytic reaction but also participates in reducing Ni^3+^ to Ni^2+^. The decrease in photocurrent performance is associated with the reduced amount of Ni^3+^. Ni^3+^ is generally regarded as an effective hole‐trapping center and a high‐valence oxidation site, thereby facilitating charge separation and interfacial redox processes. In contrast, the increased dominance of Ni^2+^ may weaken hole extraction and promote charge recombination, thereby degrading photocatalytic performance [46].

### 2.7 Interfacial Regulation by NiO_x_ in Photoelectrochemical Reactions

**FIGURE 8:**
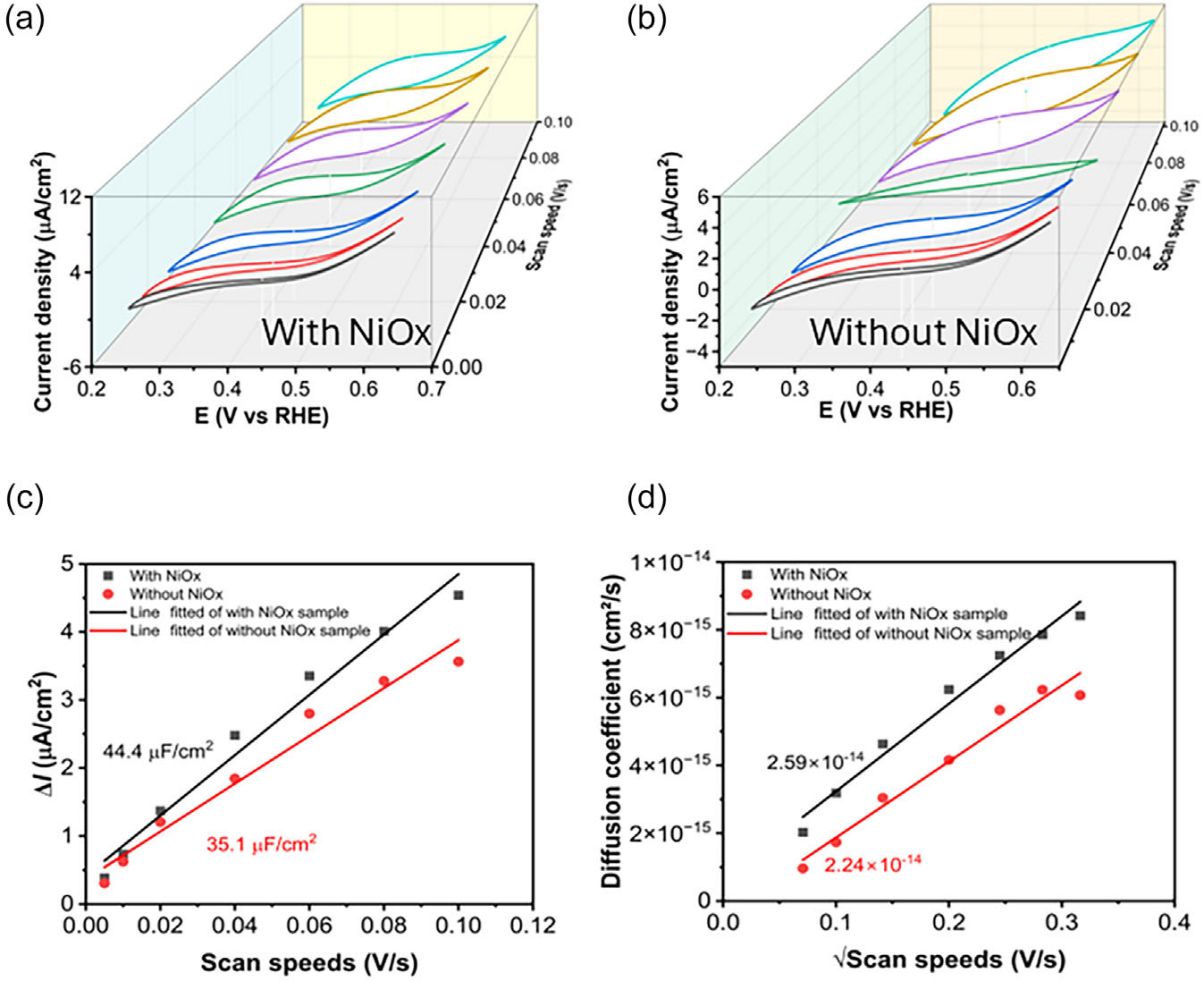
CV results under different scan speeds: (a) the NiO_*x*_‐modified electrode; (b) unmodified electrode; (c) electrochemical active surface area test; (d) Randles–Sevcik Plot.

CV measurements were conducted at various sweep speeds to assess the impact of NiO_*x*_ on electrode performance. Figure 8a,b show the results for the NiO_*x*_‐modified electrode compared to the unmodified electrode. Figure 8c illustrates the results of electrochemically active surface area measurements, showing the relationship between scan speeds and current density. For the NiO*_x_*‐modified electrode, a steeper slope is observed (44.4 μF/cm^2^) compared to the unmodified electrode (35.1 μF/cm^2^). The increased slope observed after NiO_*x*_ modification reflects a higher double‐layer capacitance, indicating that the introduction of NiO_*x*_ exposes additional surface sites that participate in interfacial electrochemical processes. The increased surface area exposes more active sites, leading to an enhanced overall catalytic current density. To clarify whether the performance variation originates from changes in reaction mechanism or surface properties, the electrochemical behavior was examined using cyclic voltammetry at different scan rates for the NiO_*x*_‐modified and unmodified electrodes (Figure 8d). The linearity of both datasets confirms that the redox process is predominantly diffusion‐controlled, as described by the Randles–Sevcik equation [47, 48]. Notably, the NiO*_x_*‐modified electrode exhibits a significantly steeper slope (2.59 × 10^−14^) compared to unmodified electrodes (2.24 × 10^−14^), indicating improved electron transfer kinetics. These enhancements are likely due to the introduction of the NiO_*x*_ layer, which promotes more efficient charge transport across the electrode–electrolyte interface and enhances electrolyte diffusion. These findings indicate that NiO_*x*_ plays a critical role in improving anodic reaction and electrolyte diffusion, leading to superior electrocatalytic performance.

### 2.8 Mechanistic Insight Into the Photoelectrochemical Reaction

**FIGURE 9:**
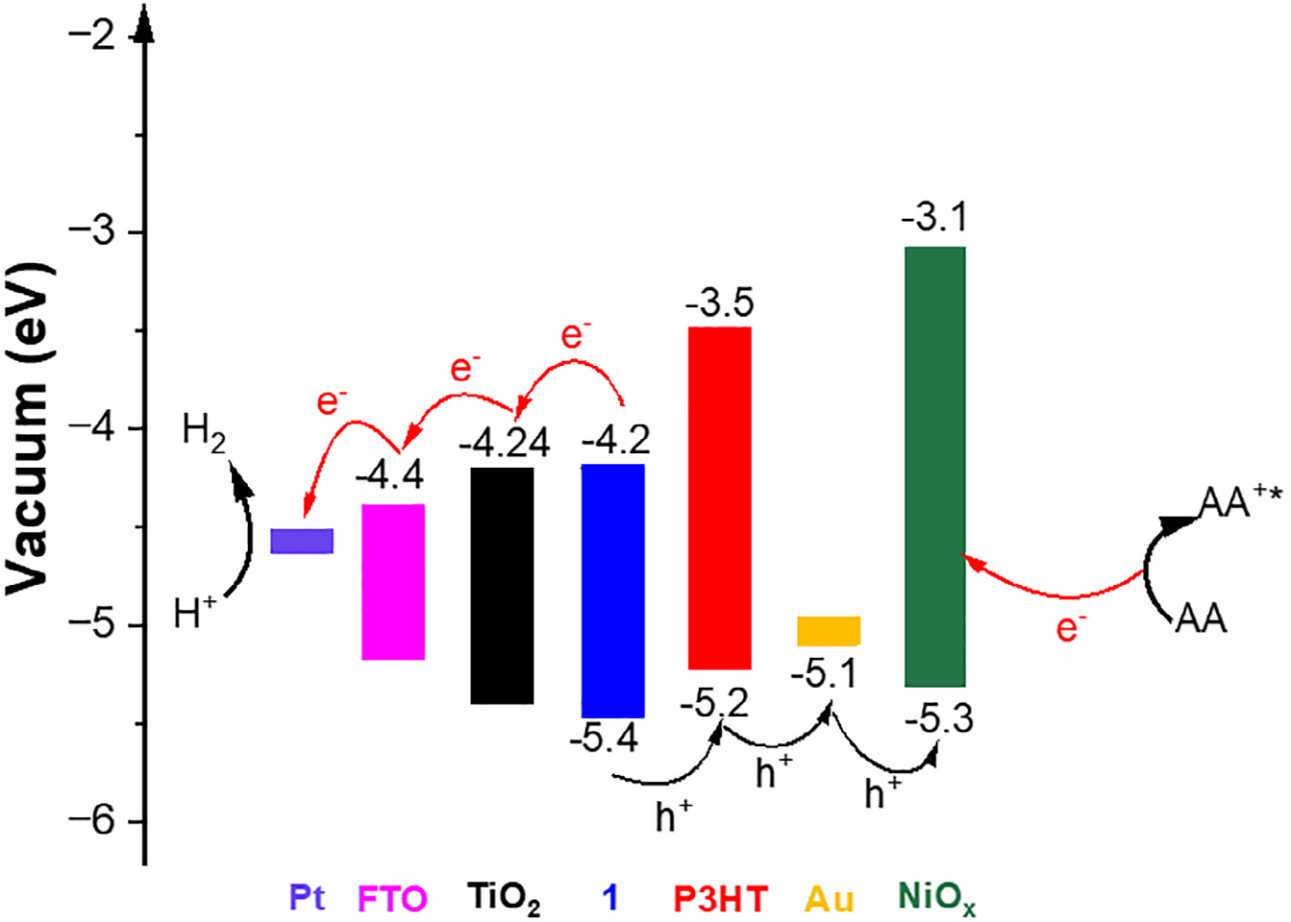
Proposed mechanism of the photocatalytic reaction.

Figure 9 illustrates the proposed charge‐transfer pathway, with the HOMO and LUMO levels measured by cyclic and differential pulse voltammetry in a 0.1 M *n*Bu_4_NPF_6_ solution in tetrahydrofuran (THF). The oxidation and reduction potentials of dye **1** were determined to be +0.89 V and −0.81 V versus the normal hydrogen electrode (NHE), respectively, corresponding to a HOMO–LUMO gap of 1.70 V (Figure S12). Moreover, the energy level of the NiO_*x*_ co‐catalyst was determined by ultraviolet photoelectron spectroscopy (UPS), revealing a valence‐band position of approximately −5.3 eV (Figure S13).

Upon illumination, dye molecule **1** is photoexcited and injects an electron into the TiO_2_ conduction band. The injected electron is then transported through the FTO substrate to the platinum counter electrode, where it participates in the hydrogen evolution reaction. Simultaneously, the photogenerated hole remaining in the dye is transferred to the P3HT hole‐transport layer. This hole‐transfer process is energetically favorable because the HOMO level of P3HT (~−5.2 eV) is more negative than that of the dye, thereby facilitating efficient hole extraction from the photoexcited sensitizer [49]. Previous studies have shown that such an energy‐level alignment promotes hole transport from dye molecules to P3HT [50]. Furthermore, efficient electronic coupling at the P3HT/Au interface has been reported, which facilitates charge extraction and hole transport across the interface [51]. The extracted hole subsequently migrates through the Au layer and reaches the surface‐deposited NiO_*x*_ co‐catalyst. In addition, interfacial electronic coupling between Au and NiO has been shown to promote charge transfer across the metal/oxide interface, thereby facilitating charge collection by the NiO_*x*_ layer [52].

At the electrode/electrolyte interface, NiO_*x*_ mediates charge transfer to the sacrificial electron donor, ascorbic acid (AA), thereby regenerating AA to its original oxidation state and sustaining the photocatalytic cycle. The favorable energy‐level alignment and interfacial electronic coupling throughout the dye/P3HT/Au/NiO_*x*_ architecture enable efficient charge separation and directional charge transport, which together contribute to the enhanced photoelectrochemical performance observed for the NiO_*x*_‐modified electrode.

## 3 Conclusion

In this study, a new FTO/TiO_2_/**1**/HTL/Au/NiO_*x*_ multilayer electrode, including the organic dye‐sensitizer **1**, is used to conduct the water‐splitting reaction under NIR irradiation. The layers include TiO_2_ as the current collector, dye‐sensitizer **1**, P3HT as a hole transfer layer, and Au as a protecting layer. NiO_*x*_ acts as both an OER cocatalyst and an interface regulator, which is fabricated by pulsed laser deposition. TEM results indicate that NiO_*x*_ exists in a low‐crystalline state, while XPS shows it to be a mixture of Ni^2+^ and Ni^3+^. After modification with NiO_*x*_, the photocurrent density is not only 1.9 times higher than that of the unmodified electrode, but it also demonstrates better light stability. The FTO/TiO_2_/**1**/HTL/Au/NiO_*x*_ electrode exhibits an IPCE of 0.01% at 850 nm, supporting the use of NIR radiation. Based on electrochemically active surface area calculations and Randles–Sevcik measurements, the NiO_*x*_‐modified electrode exhibits a significantly steeper slope than the unmodified electrode, indicating an increased active surface area and enhanced electrolyte diffusion. Overall, these results suggest that a thin layer of NiO_*x*_ not only serves as a co‐catalyst but also actively tunes the local electrochemical environment. Further rational design of NiO_*x*_ chemistry and interfacial structure is expected to advance the development of high‐performance catalysts under practical operating conditions.

## 4 Experimental Section/Methods

### 4.1 General Information

^1^H and ^13^C nuclear magnetic resonance (NMR) spectra were recorded on Bruker/Biospin AV400 (400 MHz) spectrometers. The ^1^H and ^13^C NMR chemical shifts are reported as δ values (ppm) relative to tetramethyl silane (Me4Si) as internal standard. Absorption/diffuse reflectance spectra were recorded on a Shimadzu UV–3600 spectrophotometer. Cyclic voltammetry (CV) was performed using a conventional three‐electrode configuration (platinum wire as the counter electrode and a saturated KCl Ag/AgCl reference electrode) with an electrochemical analyzer (mAUTOLAB, TYPE III, Nova 2.1.4) at a 10 mV/s scan rate in a 0.1 M nBu_4_NPF_6_ and 1 mM dye THF solution. Density functional theory (DFT) calculations were performed using Gaussian 16 C 01. XRD patterns of the samples were obtained on a high‐resolution diffractometer (SmartLab, Rigaku) with Cu Kα radiation (40 kV, 80 mA) in the 2θ range from 10° to 60° at a scan rate of 0.05°. Scanning electron microscope (SEM) images of prepared samples were obtained using a SEM (FEI, Versa 3D HiVac) at a voltage of 20 kV. X‐ray photoelectron spectroscopy (XPS) was conducted on a high‐sensitivity photoelectron spectrometer (Kratos Ultra2, Shimazu) with Mg Kα radiation. Transmission electron microscopy (TEM) analyses were performed using a JEM–ARM200F NEOARM (JEOL) operated at an accelerating voltage of 200 kV. Cross‐sectional specimens were prepared by focused ion beam milling (FIB–SEM) using an NB5000 system (Hitachi High–Technologies).

### 4.2 Thin Film Deposition

#### 4.2.1 Titanium Oxide (TiO_2_) Layer

Glass slides coated with fluorinated tin oxide (FTO) (Aldrich, 7 W/cm^2^) were pretreated, using ultrasonic cleaning in acetone and isopropanol for 15 min each, followed by ozone plasma cleaning (CUTE 1MP, Femto Science). Different thicknesses of anatase‐phase TiO_2_ films were deposited on FTO electrodes by a scrape‐coating method and calcined at 500 °C (5 °C/minute) under air for 2 h.

#### 4.2.2 Dye Sensitizer Layer

The FTO/TiO_2_ electrode was immersed in a THF solution containing **1** (1 × 10^−4^ M) at room temperature for 24 h in the dark. A loading amount (0.53 mmol/cm^2^) is estimated by the difference between the pre‐ and post‐load absorption spectra. Details of the synthetic procedure for **1** are provided in the Supporting Information.

#### 4.2.3 Hole Transporting Layer (HTL)

The HTL stock solution was prepared by dissolving 5.0 mg of poly‐3‐hexyl thiophene (P3HT) in 1.0 mL of THF solution. The HTL solution was spin‐coated (20 μL, 3000 rpm, 10 s) on the FTO/TiO_2_/**1** electrode. Then, it was dried at room temperature overnight.

#### 4.2.4 Au Layer

A 100 nm thick gold layer was deposited onto the FTO/TiO_2_/**1**/HTL layers by the thermal vacuum deposition method to fabricate the FTO/TiO_2_/**1**/HTL/Au device structure.

#### 4.2.5 Co‐Catalyst Layer

The NiO_*x*_ thin films used in this work were grown using pulsed laser deposition (PLD). A 248 nm (20 ns pulse width) KrF excimer laser (Lambda Physik LPX 300) was used with a home‐made chamber. A laser fluence of ca. 1.7 J cm^−2^, spot size of 0.013 cm^2^, and a repetition rate of 10 Hz. A rod‐style target was fabricated in‐house by pressing and sintering NiO powder (99.99%, Sigma–Aldrich). Films were deposited on FTO/TiO_2_/**1**/HTL/Au electrodes (10.0 mm × 0.5 mm × 0.5 mm) with a substrate‐to‐target distance fixed at 50 mm under 0.45 millibar oxygen atmosphere. The thickness of NiO_*x*_ was calculated from the relationship between PLD deposition time and the actual TME measurements (4.8 Å/s).

### 4.3 Photocurrent Measurement

A conventional three‐electrode configuration, composed of FTO/TiO_2_/**1**/HTL/Au/NiO_*x*_ as the working electrode, a platinum wire as the counter electrode, and Ag/AgCl as the reference electrode, was employed. All measurements were performed in deionized water solutions containing 0.1, 0.2, and 0.3 M Na_2_SO_4_ and 0.4, 0.6, and 0.8 M ascorbic acid (AA) as the supporting electrolyte, with the pH adjusted using NaOH. The photocurrent properties were obtained under irradiation with a 300 W Xe lamp using a long‐pass filter (Edmund optics, > 420 nm filter).

#### 4.3.1 Incident Photon‐to‐Current Efficiencies (IPCE)

IPCE were determined for FTO/TiO_2_/**1**/HTL/Au/NiO_*x*_ electrodes at wavelengths of 650 ± 5 nm, 700 ± 5 nm, 750 ± 5 nm, 800 ± 5 nm, and 850 ± 5 nm that were obtained by the application of various through‐band‐pass filters. The IPCE was calculated according to Equation (1):

(1) $$\mathrm{IPCE}=\frac{N_{c}}{N_{p}}\times 100\%=\frac{J\times \lambda \times q}{I\times h\times c}\times 100\%$$

where *J* (W m^−2^) represents photocurrent density, *λ* (m) is the wavelength, *q* (C) is the elementary charge, *I* (W m^−2^) is the light intensity, *h* (J s) is the Planck constant, and *c* (m s^−1^) is the speed of light.

#### 4.3.2 Electrochemically Active Surface Areas (ECSA)

ECSA of electrodes were calculated according to the following Equation (2)

(2) $$\mathrm{ECSA}=\frac{\Delta J}{v}=\frac{J_{t}-J_{d}}{v}$$

where ECSA (*μ*F cm^−2^) represents the electrochemically active surface area, *J*_t_ (m) is the top photocurrent density at 0.41 V, and *J*_d_ (m) is the down photocurrent density at 0.41 V.

The Randles–Sevcik plots were calculated using Equations (3) and (4):

(3) $$i_{p}=\left(2.69\times 10^{5}\right)\times n^{\frac{3}{2}}\times A\times D^{\frac{1}{3}}\times C\times v^{\frac{1}{2}}$$

(4) $$Q=\left(2.69\times 10^{5}\right)\times n^{\frac{3}{2}}\times D^{\frac{1}{3}}\times C\times v^{\frac{1}{2}}$$

where *i*_p_ (A) represents the peak current, *C* (mol/cm^3^) is the concentration of electrolytes, *n* is the number of electrons transferred, *v* (V/s) is the scan rate, *A* (cm^2^) is the electrode area, *D* (cm^2^/s) is the diffusion coefficient, and *Q* (A/cm^2^) is the total charge (≈CV area).

## Funding

This work was supported by the Japan Science and Technology Agency (JPMJAP2332), Schweizerischer Nationalfonds zur Förderung der Wissenschaftlichen Forschung (204103 and 216196).

## Supporting information

Supplementary Material

## Data Availability

The data that support the findings of this study are available from the corresponding author upon reasonable request.
