# Supplementary material for: Interfacial Regulation by a NiO x Overlayer Enables Enhanced Near‐Infrared Photoelectrochemical Water Splitting
Source: Small Sci. 2026 Jul 23;6(7):e70346. doi: 10.1002/smsc.70346 (PMC13410517; doi:10.1002/smsc.70346)
Supplement: Supplementary file 1 — Supplementary Material [file SMSC-6-e70346-s001.pdf]

Supporting Information

Interfacial Regulation by a NiO_x Overlayer Enables Enhanced Near-Infrared Photoelectrochemical Water Splitting

Xiao−Feng Shen [1,2*] | Kyle J. Stephens [2,3] | Dengyao Yang [1] | Nick A. Shepelin [2] | Kuan−Ting Wu [1] | Kazuto Hatakeyama [4] | Daniele Pergolesi [2,5] | Shintaro Ida [4] | Motonori Watanabe [1,6*] | Thomas Lippert [1,2,3*]

1. International Institute for Carbon−Neutral Energy Research, Kyushu University, Fukuoka, Japan | 2. Center for Neutron and Muon Sciences, Paul Scherrer Institute, 5232 Villigen PSI, Switzerland | 3. Department of Chemistry and Applied Biosciences, ETH Zürich, 8093 Zürich, Switzerland | 4. Institute of Industrial Nanomaterials (IINa), Kumamoto University, Kumamoto, Japan | 5. PSI Center for Energy and Environmental Sciences, Paul Scherrer Institute, 5232 Villigen PSI, Switzerland | 6. Hydrogen Institute for Sustainability, Kyushu University, Fukuoka, Japan

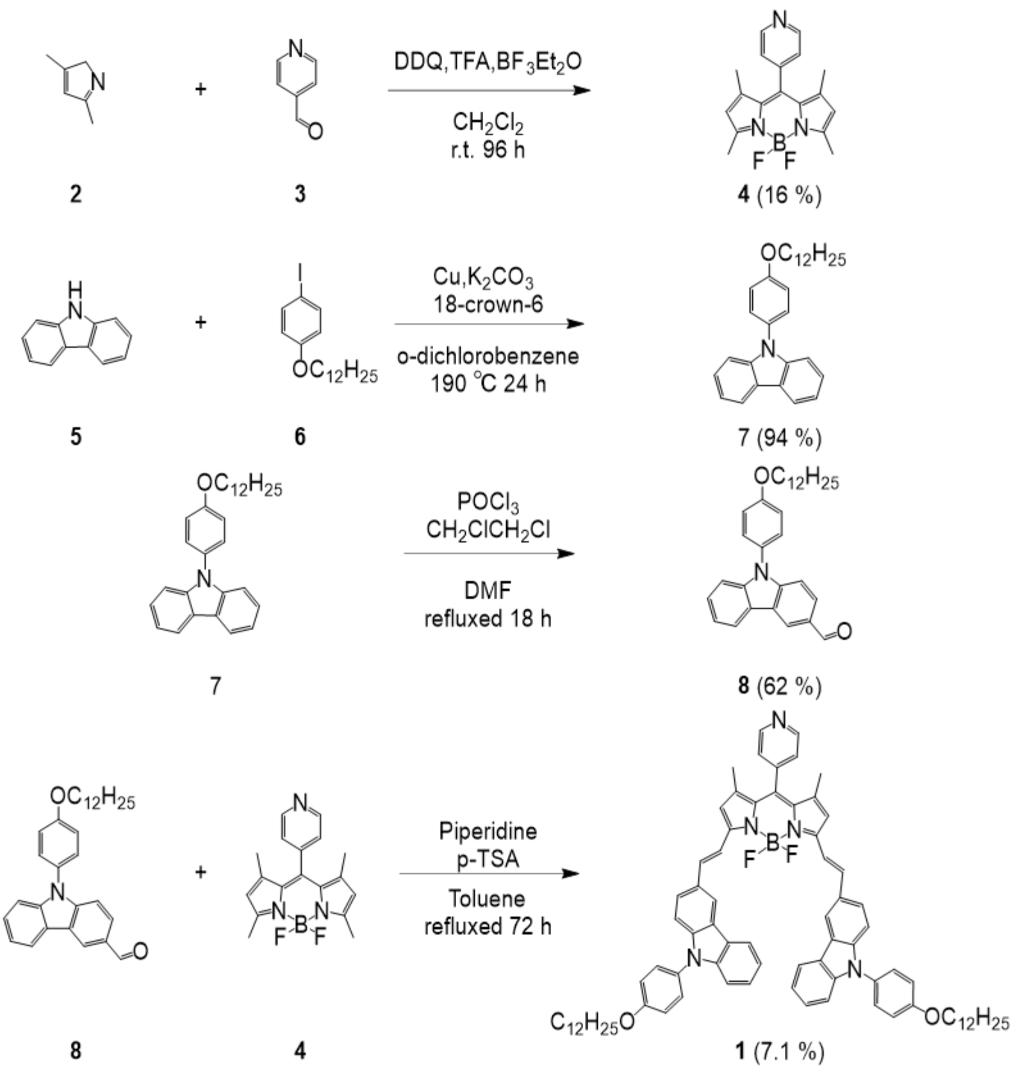

Scheme. **1** Synthesized route of **1**.

1-1 Synthesis of 5,5-difluoro-1,3,7,9-tetramethyl-10-(pyridin-4-yl)-5H-4l4,5l4-dipyrrolo[1,2-c:2',1'-f] [1,3,2]diazaborinine ***4***

A solution of 3,5-dimethyl-2H-pyrrole **2** (4.9 g,51.5 mmol) and 4-Pyridinecarboxaldehyde **3** (2.3 g, 21.5 mmol) in dichloromethane (375 mL) under a nitrogen atmosphere. After added Trifluoroacetic acid (TFA, 8 drops), the solution was heated to reflux for 96 h. Then, 2,3-dichloro-5,6-dicyano-p-benzoquinone (DDQ, 3.2 g,14.1 mmol) was added, and the mixture was stirred for 1 h in ice bath. NEt_3 (20.9 mL, 150 mmol), and $BF_3 \cdot Et_2O$ (27.8 mL, 202.3 mmol) was added and stirred for 98 h at room temperature. After the reaction, the mixture was concentrated in vacuo. The crude product was purified by a silica gel chromatograph eluted with CH_2Cl_2/ethyl acetate (50:1) to give compound **3** (1.1 g, 16 %) as dark green solids.

^{1}H (400 MHz, $CDCl_3$) 1.41 (s, 6H), 2.56 (s, 6H), 6.01 (s,2H), 7.32 (d, J=6.0 Hz, 2H), 8.79 (d, J=5.9 Hz, 2H).

1-2 Synthesis of 9-(4-(dodecyloxy)phenyl)-9H-carbazole ***7***

A solution of 9H-carbazole **5** (2.0 g,12 mmol), **6** (3.9 g,10.0 mmol), copper (1.68 g,26 mmol), 18-crown-6-ether (0.3 g, 1.14 mmol) and Potassium carbonate (12.4 g, 90 mmol) in o-dichlorobenzene (50 mL). The solution was heated to reflux for 24 h. After the reaction, the mixture was concentrated in vacuo. The crude product was purified by a silica gel chromatograph eluted with hexane to give compound **7** (4.0 g, 94 %) as white solids.

^{1}H (400 MHz, $CDCl_3$) 0.86-0.90 (m, 3H), 1.22-1.47 (m, 16H), 1.46-1.55 (m, 2H), 1.80-1.89 (m, 2H), 4.04 (t, J = 6.5 Hz, 2H), 7.08 (d, J = 8.9 Hz, 2H), 7.27 (d, J = 7.8 Hz, 2H) , 7.32 (d, J = 8.1 Hz, 2H) , 7.38 (d, J = 7.0 Hz, 2H) ,7.42 (d, J = 9.6 Hz, 2H), 8.13 (d, J = 7.7 Hz, 2H).

1-3 Synthesis of 9-(4-(dodecyloxy)phenyl)-9H-carbazole-3-carbaldehyde ***8***

A solution of DMF (1 mL) and 1,2-dichloroethane (3 mL) was stirred in ice bath under a nitrogen atmosphere. $POCl_3$ (1 mL) was added and stirred for 0.5 h at room temperature, until the color of solution changed colorless to pale yellow. Then, precursor **7** (0.3 g,1.0 mmol) was added. The solution was heated to reflux for 18 h. After the reaction, the mixture was concentrated in vacuo. The crude product was purified by a silica gel chromatograph eluted with CH_2Cl_2/hexane (2:1) to give compound **8** (0.3 g, 66 %) as white solids.

^{1}H (400 MHz, $CDCl_3$) 0.86-0.90 (m, 3H), 1.22-1.39 (m, 16H), 1.47-1.58 (m, 2H), 1.82-1.90 (m, 2H), 4.06 (t, J = 6.5 Hz, 2H), 7.12 (d, J = 8.8 Hz, 1H), 7.31-7.50 (m, 5H) ,7.93 (dd, J = 1.5, 7.0 Hz, 1H), 8.20 (d, J = 7.7 Hz, 1H) , 10.1 (s,1H).

1-4 Synthesis of ***1***

A solution of precursor **8** (1.1 g, 2.4 mmol) and **4** (0.2 g, 0.6 mmol) in toluene (50 mL) at room temperature. Then, p-TSA (0.1 g, 5.2 mmol) and piperidine (1.5 ml) was added. The solution was heated to reflux for 90 h. After the reaction, the mixture was concentrated in vacuo. The crude product was purified by a silica gel chromatograph eluted with CH_2Cl_2 to give compound **1** (0.3 g, 42 %) as dark green solids.

^{1}H (400 MHz, CD_2Cl_2) δ = 0.85-0.90 (m, 6H), 1.19-1.42 (m, 34H), 1.37-1.58 (m, 8H), 1.80-1.89 (m, 4H), 4.08 (t, J = 6.5 Hz, 4H), 6.19-6.22 (m, 4H) ,6.78 (s, 2H), 6.71-6.77(m,4H), 7.33-7.49 (m, 14H), 7.55(s,1H), 7.59(s,1H), 7.75-7.79 (m, 3H), 7.81(s, 1H), 8.27 (d, J = 7.7 Hz, 2H), 8.44 (s, 2H), 8.79 (d, J = 5.1 Hz, 2H).

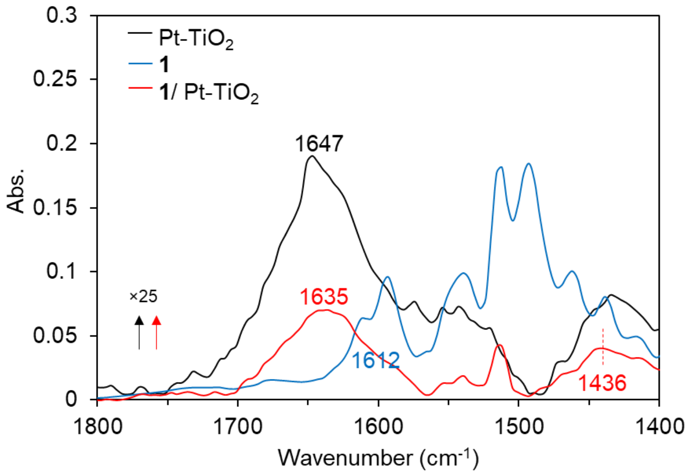

Figure. S1 ATR-IR result of **1**, Pt-TiO_2 and **1**/Pt-TiO_2.

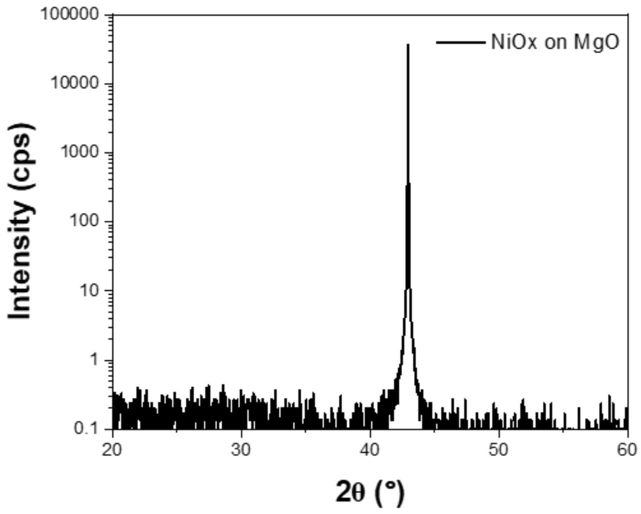

Figure. S2 XRD result of NiO_x on MgO sample.

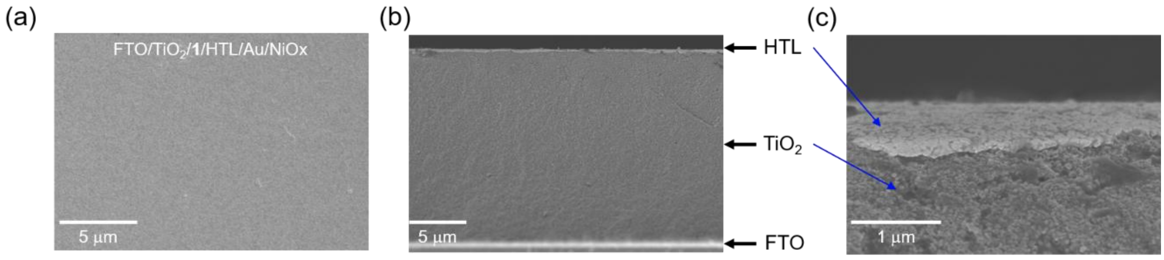

Figure. S3 SEM images of the fresh electrode: (a) top view; (b) side view; (c) cross-section view.

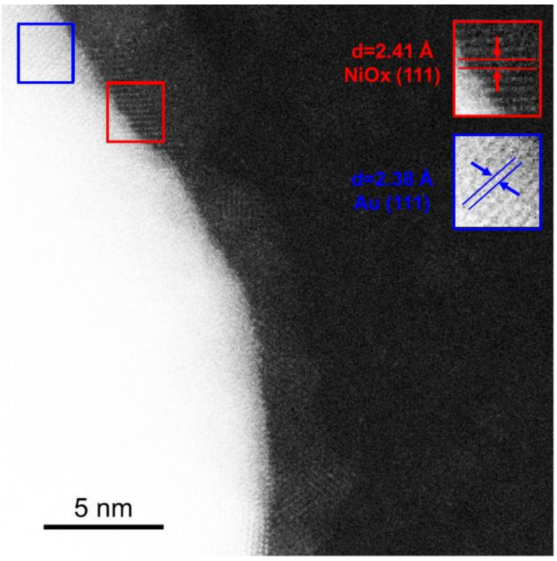

Figure. S4 The TEM image of the fresh electrode.

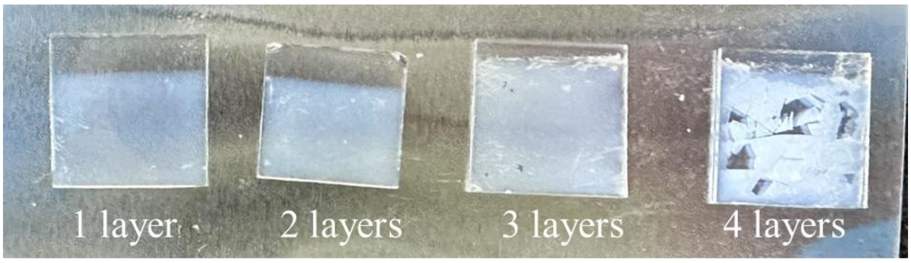

Figure. S5 Photographs of electrodes with different TiO_2 thicknesses.

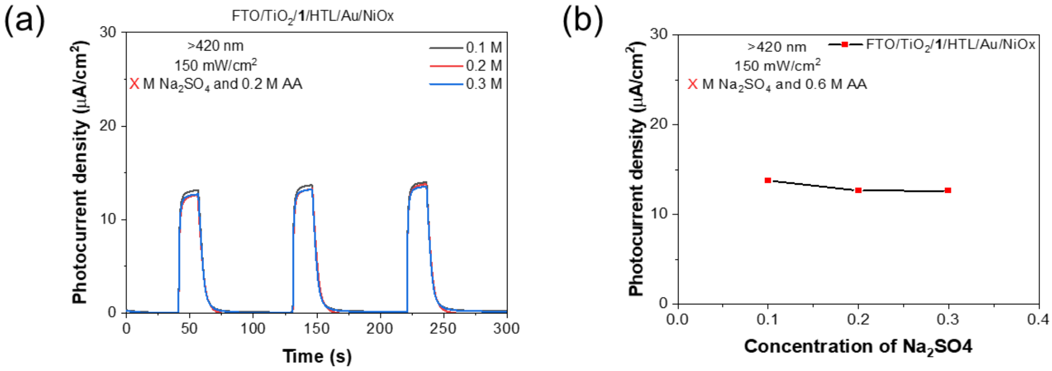

Figure. S6 (a) Photocurrent density of different Na_2SO_4 concentrations; (b) the relationship between Na_2SO_4 concentrations and photocurrent performances.

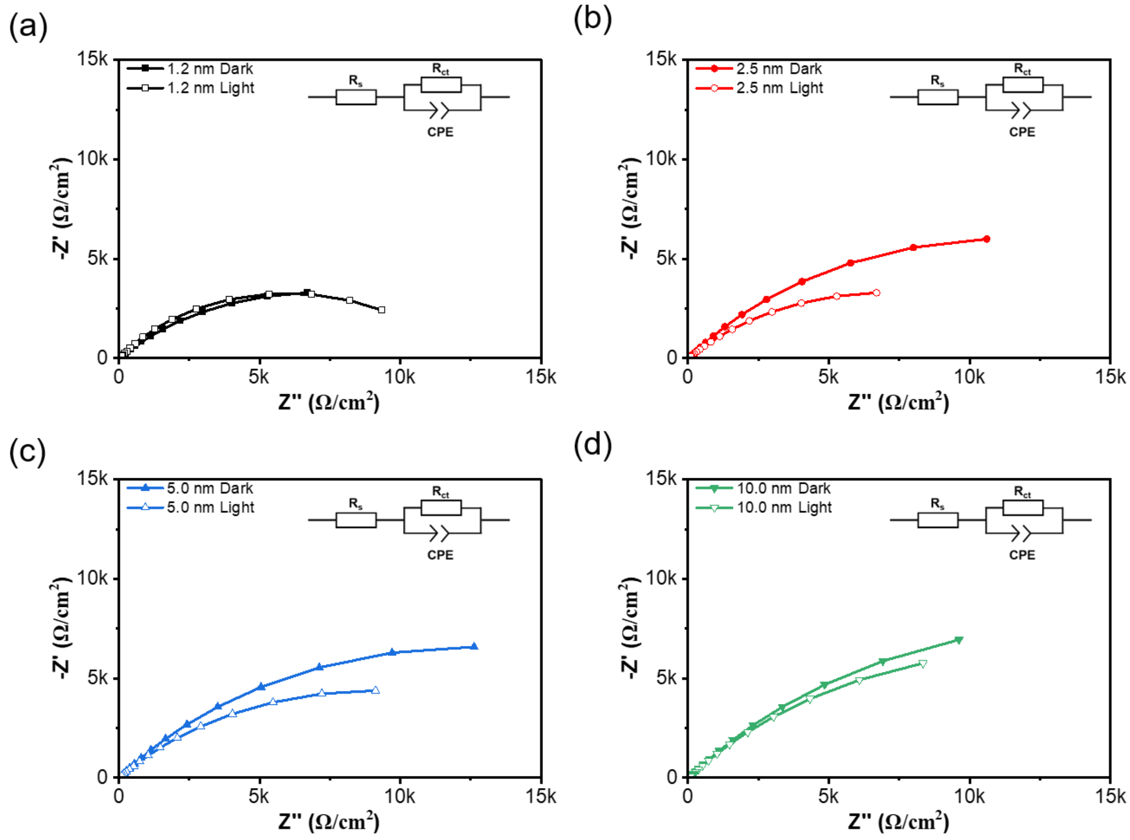

Figure. S7 Impedance spectra of **dye**/TiO_2 (0.15 W cm^{-2}, >420 nm, 0.6 M ascorbic acid aq., 0.1 M Na_2SO_4, pH 4.0).

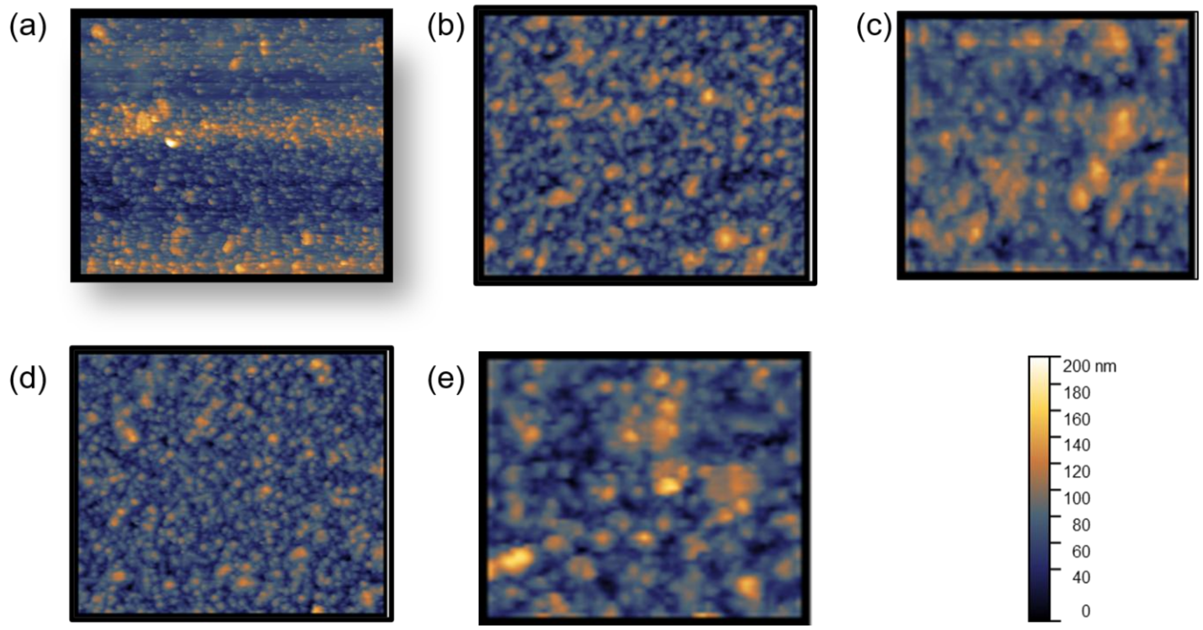

Figure. S8 AFM images of each samples: (a) without NiOx sample, (b) with 1.2 nm NiO_x sample, (c) with 2.5 nm NiO_x sample, (d) with 5.0 nm NiO_x sample, (e) with 10.0 nm NiO_x sample.

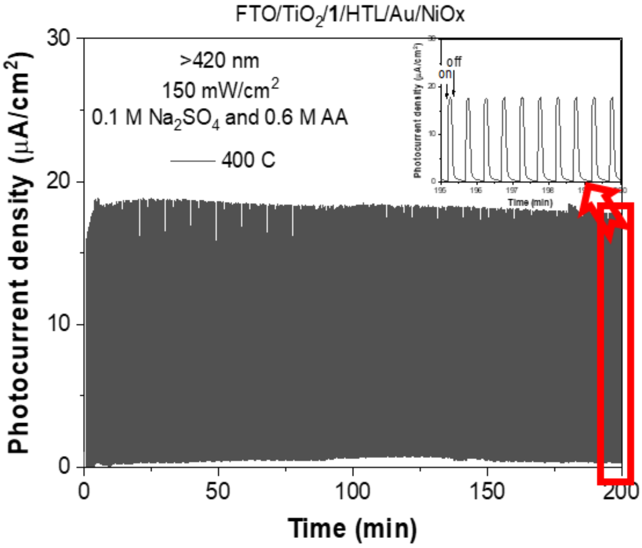

Figure. S9 Photocurrent density measurements for 400 light-on/off cycles.

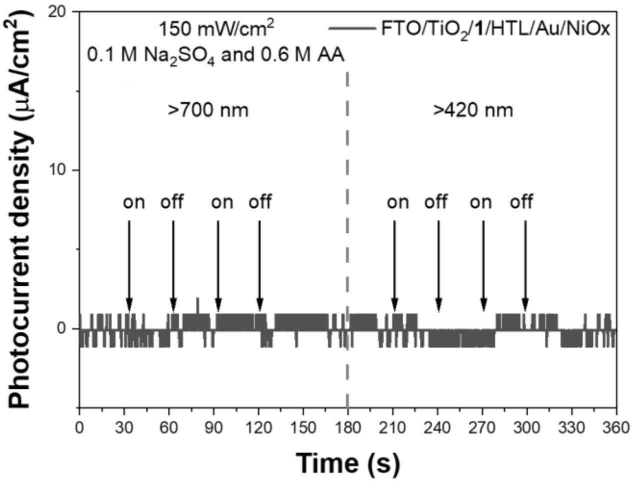

Figure. S10 Photocurrent density measurements for FTO/TiO_2/Au electrode.

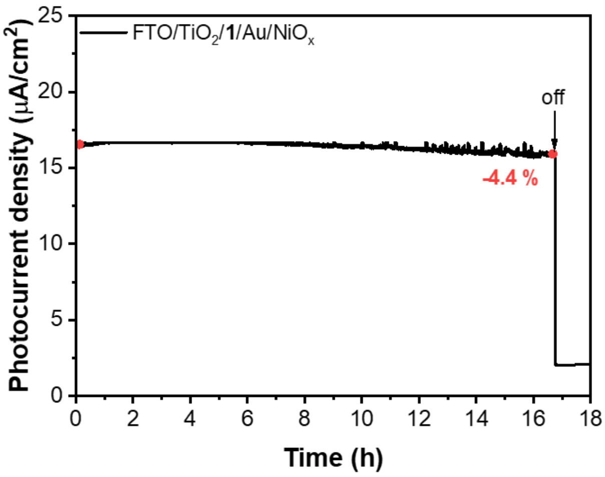

Figure. S11 Long–term light stability result of FTO/TiO_2/**1**/Au/NiO_x for 16.5 h.

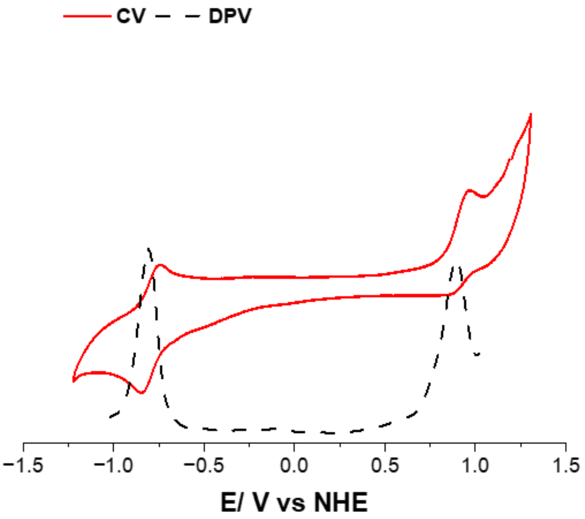

Figure. S12 Cyclic and differential pulse voltammetry results of **1** in THF solution.

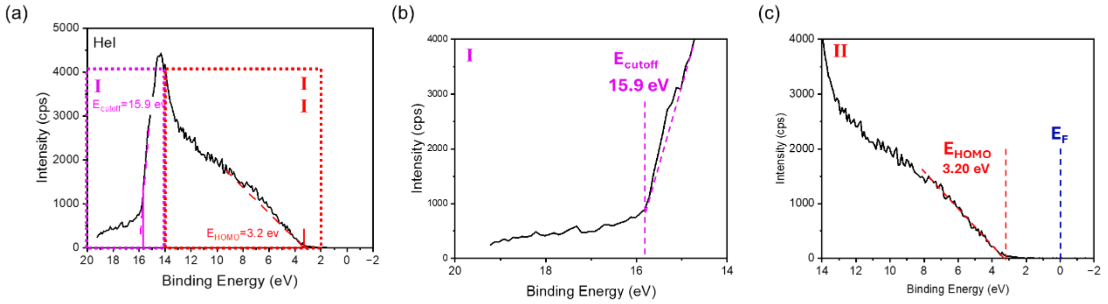

Figure. S13 UPS results of NiO_x-MgO sample: (a) overview; (b) Enlarge view between 14-20 eV; (c) Enlarge view between -2-14 eV.

Table S1. Impedance results of different thicknesses NiO_x

	R_s (Ω)		R_{ct} (Ω)	
	Dark	Light	Dark	Light
1.2 nm	30.27	30.67	14149	11913
2.5 nm	37.78	30.27	22994	14149
5.0 nm	25.15	25.66	25353	18279
10.0 nm	45.83	47.18	29919	25285

Table S2. Summary of NIR-active photocatalytic systems with the absorption wavelength larger than 700 nm and the related photocatalytic stability

Photocatalytic systems	Irradiation light	decomposition rate	Ref.
g-C_3N_4 quantum dots/ultrathin Bi_2WO_6 nanosheets	λ > 700 nm	-5% after 4 h	[1]
Ag_2S/Ag_3PO_4	λ > 760 nm	-3% after 6 h	[2]
$Bi_2WO_6/Cu_{1.8}Se$	λ > 800 nm	-20% after 9h	[3]
Sillenite-type $Bi_{12}MnO_{20}$	100 W NIR LED with the peak centered at 815 nm	-8% after 8 h	[4]
Vacancy-rich monolayer BiO_{2-x}	770 nm < λ < 860 nm	-16% after 25 h	[5]
FTO/TiO_2/**1**/HTL/Au/NiO_x	λ > 850 nm	-4.4% after 16.5 h	This work

References

1. Zhang, M., Zhang, Y.et al., "Ultrathin Bi_2WO_6 Nanosheets Loaded G-C_3N_4 Quantum Dots: A Direct Z-Scheme Photocatalyst with Enhanced Photocatalytic Activity towards Degradation of Organic Pollutants under Wide Spectrum Light Irradiation." *Journal of Colloid and Interface Science*, **539** (2019), 654–664. https://doi.org/10.1016/j.jcis.2018.12.112.

2. Tian, J., Yan, T.et al., "Anion-Exchange Synthesis of Ag_2S/Ag_3PO_4 Core/Shell Composites with Enhanced Visible and NIR Light Photocatalytic Performance and the Photocatalytic Mechanisms." *Applied Catalysis B: Environment and Energy*, **207** (2017), 566–578. https://doi.org/10.1016/j.apcatb.2017.03.022.
3. Qiao, L. N., Wang, H. C.et al., "Generation of Hydrogen under Visible Light Irradiation with Enhanced Photocatalytic Activity of $Bi_2WO_6/Cu_{1.8}Se$ for Organic Pollutants under Vis-NIR Light Reign." *Journal of the American Ceramic Society*, **101** (2018), 3015–3025. https://doi.org/10.1111/jace.15433.
4. Wu, X., Li, M.;et al., "A Sillenite-Type $Bi1_2MnO_{20}$ Photocatalyst: UV, Visible and Infrared Lights Responsive Photocatalytic Properties Induced by the Hybridization of Mn 3d and O2p Orbitals." *Applied Catalysis **B**: Environment and Energy*, **219** (2017), 132–141. https://doi.org/10.1016/j.apcatb.2017.07.025.
5. Li, J., Wu, X.et al., "Rich Monolayer BiO_{2-x} as a Highly Efficient UV, Visible, and Near-Infrared Responsive Photocatalyst." *Angewandte Chemie International Edition*, **130** (2018), 500–504. https://doi.org/10.1002/ange.201708709.
